# Clinical and Biochemical Function of Polymorphic *NR0B1* GGAA-Microsatellites in Ewing Sarcoma: A Report from the Children's Oncology Group

**DOI:** 10.1371/journal.pone.0104378

**Published:** 2014-08-05

**Authors:** Michael J. Monument, Kirsten M. Johnson, Elizabeth McIlvaine, Lisa Abegglen, W. Scott Watkins, Lynn B. Jorde, Richard B. Womer, Natalie Beeler, Laura Monovich, Elizabeth R. Lawlor, Julia A. Bridge, Joshua D. Schiffman, Mark D. Krailo, R. Lor Randall, Stephen L. Lessnick

**Affiliations:** 1 Sarcoma Services, Department of Orthopedic Surgery, University of Utah, Salt Lake City, Utah, United States of America; 2 Center for Children's Cancer Research, Huntsman Cancer Institute, University of Utah, Salt Lake City, Utah, United States of America; 3 Department of Preventive Medicine, Keck School of Medicine, University of Southern California, Los Angeles, California, United States of America; 4 Department of Human Genetics and Eccles Institute of Human Genetics, University of Utah, Salt Lake City, Utah, United States of America; 5 Division of Oncology, The Children's Hospital of Philadelphia, University of Pennsylvania, Philadelphia, Pennsylvania, United States of America; 6 Children's Oncology Group Biopathology Center, The Research Institute at Nationwide Children's Hospital, Columbus, Ohio, United States of America; 7 Departments of Pediatrics and Pathology, University of Michigan, Ann Arbor, Michigan, United States of America; 8 Department of Pathology and Microbiology, University of Nebraska Medical Center, Nebraska Medical Center, Omaha, Nebraska, United States of America; 9 Division of Pediatric Hematology/Oncology, University of Utah, Salt Lake City, Utah, United States of America; Johns Hopkins University, United States of America

## Abstract

**Background:**

The genetics involved in Ewing sarcoma susceptibility and prognosis are poorly understood. EWS/FLI and related EWS/ETS chimeras upregulate numerous gene targets via promoter-based GGAA-microsatellite response elements. These microsatellites are highly polymorphic in humans, and preliminary evidence suggests EWS/FLI-mediated gene expression is highly dependent on the number of GGAA motifs within the microsatellite.

**Objectives:**

Here we sought to examine the polymorphic spectrum of a GGAA-microsatellite within the *NR0B1* promoter (a critical EWS/FLI target) in primary Ewing sarcoma tumors, and characterize how this polymorphism influences gene expression and clinical outcomes.

**Results:**

A complex, bimodal pattern of EWS/FLI-mediated gene expression was observed across a wide range of GGAA motifs, with maximal expression observed in constructs containing 20–26 GGAA motifs. Relative to white European and African controls, the *NR0B1* GGAA-microsatellite in tumor cells demonstrated a strong bias for haplotypes containing 21–25 GGAA motifs suggesting a relationship between microsatellite function and disease susceptibility. This selection bias was not a product of microsatellite instability in tumor samples, nor was there a correlation between *NR0B1* GGAA-microsatellite polymorphisms and survival outcomes.

**Conclusions:**

These data suggest that GGAA-microsatellite polymorphisms observed in human populations modulate EWS/FLI-mediated gene expression and may influence disease susceptibility in Ewing sarcoma.

## Introduction

Ewing sarcoma is a prototypical chromosomal translocation-associated malignancy, in which virtually all cases harbor a balanced somatic translocation fusing the *EWSR1* gene (EWS) to a member of the (E- twenty six) ETS-family of transcription factors, most commonly *FLI1* (FLI) [1], [2]. In fact, EWS/FLI and related EWS/ETS fusions are considered pathognomonic for the diagnosis of Ewing sarcoma. The EWS/FLI chimera product is a potent oncogenic transcription factor, characterized by fusion of a transcriptional-regulatory domain of EWS to the DNA binding domain of FLI [2]. EWS/FLI is considered the master-regulator of oncogenesis in Ewing sarcoma, regulating numerous critical gene targets necessary for oncogenic transformation [3], [4].

Genome-wide localizations studies utilizing ChIP-seq and ChIP-chip strategies have identified many direct EWS/FLI targets. A remarkable observation derived from these studies was a previously unrecognized affinity of the EWS/FLI chimera for a repetitive GGAA-microsatellite element embedded within promoter/enhancer regions of numerous upregulated gene targets [5]–[8]. Forty to fifty percent of genomic EWS/FLI binding sites are associated with these GGAA-microsatellites [7] and EWS/FLI-mediated DNA binding and gene expression is dependent on these repetitive GGAA response elements [5], [8], [9]. These findings collectively demonstrate an unprecedented link between microsatellite DNA and transcriptional dysregulation in Ewing sarcoma.

Microsatellite DNA tracts represent ∼3% of the human genome and are commonly located in non-coding extra-genic regions [10]. The repetitive nature and non-coding position of these elements allows microsatellite DNA to experience a higher baseline mutational rate than coding DNA. Consequently, these genetic elements are highly polymorphic at both an individual and population level [11]. Recently it has been shown that the GGAA-microsatellites within two critical upregulated EWS/FLI-target genes (*NR0B1* and *CAV1*) are highly polymorphic in healthy human subjects. Notably, significant length-dependent differences were observed comparing the *NR0B1* GGAA-microsatellite in white European and African populations [12]. This is significant as the incidence of Ewing sarcoma is 10-fold less in African populations compared to white Europeans, irrespective of geographic location, suggesting a likely genetic influence [13]. Furthermore, *NR0B1* is among the most highly upregulated EWS/FLI targets and is essential for oncogenesis in Ewing sarcoma [6], [14].

Initial studies characterizing the biochemical properties of these GGAA-microsatellite response elements demonstrated EWS/FLI DNA binding and subsequent transcriptional activation is highly dependent on the number of GGAA motifs within in the microsatellite: A minimum of 4 GGAA motifs is required for initial DNA binding, and gene expression markedly increases in a length-dependent manner with additional GGAA motifs [5], [9], [15]. Importantly, these early biochemical studies only characterized the relationship of EWS/FLI DNA binding and gene expression over a small and narrow range of 1–11 GGAA motifs. It remains unclear how the substantially larger spectrum of GGAA-microsatellite polymorphisms, observed in human populations influences EWS/FLI-mediated transcriptional activity. The goal of the present study was to characterize the polymorphic spectrum of the *NR0B1* GGAA-microsatellite in Ewing sarcoma tumors, define the biochemical properties of these GGAA length polymorphisms and to determine whether clinical outcomes are influenced by variations in these genetic elements.

## Materials and Methods

### Ethics statement

This study was approved by the University of Utah, Office for Research Integrity and Compliance prior to commencement. All patients enrolled in the Children's Oncology Group (COG) study AEW0031 (or legal guardians) provided written informed consent prior to study enrollment, which included the use of patient samples and tissues for molecular studies. All patient samples analyzed in the present study were de-identified and re-identification of samples was strictly reserved for the COG Statistics and Data Center to perform the appropriate clinical outcomes analysis. This study was carried out in accordance with the Declaration of Helsinki.

### Patient samples

Ewing sarcoma tissue samples were obtained from the Biopathology Center (Columbus, OH), which serves as the specimen bank for the Children's Oncology Group. Patient demographics such as age, sex and race were self-reported by the patient (or legal guardians) at the time of study enrollment. Patients were instructed to identify their race as Caucasian, African American, Asian, Pacific Islander, American Indian or other. DNA from these tissue samples was extracted from OCT embedded tissue blocks or snap frozen tumors courtesy of Dr. Julie Bridge (University of Nebraska Medical Center, Omaha, NE). Approximately 20 nanograms of extracted genomic DNA also were commercially amplified using Qiagen's REPLI-g service (Qiagen Genomic Services, Hilden, Germany) for whole genomic amplification (WGA).

A second cohort of 20 Ewing sarcoma tumor samples and matching bone marrow aspirates collected at our local institute were also obtained. Tissues were stored in FFPE blocks and 5-micron scrolls were cut from each block in triplicate. DNA was extracted using the RecoverAll^Tm^ Total Nucleic Acid Isolation Kit (Life Technologies, Carlsbad, CA).

### PCR sequencing

Forward and reverse primers, flanking the *NR0B1* GGAA-microsatellite loci were designed using promoter sequences obtained from the University of California Santa Cruz Human Genome Browser (http://genome.ucsc.edu/cgi-bin/hgGateway). All polymerase chain reaction (PCR) amplifications were performed using Pfx polymerase (Invitrogen, Grand Island, NY) in accordance with established laboratory protocols for microsatellite DNA. Each 25 mL PCR reaction consisted of 40–80 ng of genomic DNA, 0.3 mM of forward and reverse primers, 1U of Pfx polymerase, 0.8 mM of each deoxyribonucleotide triphosphate, 1X Pfx buffer and 1X Pfx enhancer solution. PCR products were subcloned into competent DH5a E. coli, with each bacterial colony representing an individual PCR-amplification clone. Twelve clones for each subject were selected and commercially sequenced (Beckman Coulter Genomics, Danvers, MA).

### PCA analysis

*NR0B1* GGAA-microsatellite sequence data for all samples were aligned using clustalx2. Because computational methods perform poorly on repetitive sequence, manual refinement was also necessary. Alignments in the repetitive regions were anchored on eight different single nucleotide adenosine residues that partition the contiguous GGAA repeats from the largest observed GGAA-microsatellite. The first 29 and last 50 bases of each raw sequence file were considered non-repetitive. For each contiguous GGAA segment, the number of GGAA repeats was counted and the count of the base differences between each non-repetitive region and the consensus sequence was determined (gap weight = 0.25). The pairwise distances between haplotypes were calculated as the squared Euclidian distance based on the 11 variable segments. Principal components analysis was performed using the MATLAB software package (The Mathworks, Natick, MA).

### Luciferase Experiments

The pGL3 promoter luciferase vector (Promega, Madison, WI) was used for all experimental and control conditions. Human-derived *NR0B1* GGAA-microsatellite polymorphisms or synthetic GGAA constructs were cloned directly upstream of the SV40 minimal promoter element. 293EBNA cells were transfected with experimental reporter plasmid constructs or control plasmids, the Renilla plasmid and plasmids with and without EWS/FLI cDNA. Firefly luciferase activity was normalized to *Renilla* luciferase activity to control for transfection efficiency. Each experimental condition was performed in triplicate. Two-tailed Student's *t* tests were used for statistical comparisons.

### Quantitative reverse-transcriptase polymerase chain reaction

Total RNA from established Ewing sarcoma cell lines (A673, COG-E-352, RDES, TC71 and SKES1) [16]–[19] and 293EBNA cells was amplified and detected using SYBR green fluorescence for quantitative analysis [20]. Normalized fold *NR0B1* expression in each of the Ewing sarcoma cell lines was calculated by determining the fold-change of each cell line relative to 293EBNA cells (negative control), with the data in each condition normalized to an internal housekeeping control gene *RPL21*. All experiments were performed in triplicate. Two-tailed Student's *t* tests were used for statistical comparisons.

### Microarray data

Total RNA was extracted from fresh-frozen tumor specimens using miRNAeasy columns (Qiagen). RNA was then processed and hybridized to Affymetrix HuEx 1.0 arrays in the Genome Core at Children's Hospital Los Angeles according to standard Affymetrix protocols. Data for core probeset regions were quantile-normalized using robust multi-chip averaging in the Partek Genomics Suite software platform (Partek, St. Louis, Mo). *NR0B1* transcript level data were derived from normalized exon data using median summarization. Two-tailed Student's *t* tests were used for statistical comparisons.

### Clinical outcomes analysis

Biological specimens were obtained from tissue submitted with consent for banking from eligible patients enrolled on COG study AEWS0031 [21]. The primary objective of that trial was to compare two chemotherapy regimens with respect to risk for an analytic event (EFS). Enrollment of 4.5 years with an additional year of follow-up provided for the detection of a hazard ratio (HR) of 0.64 in the failure rate with a probability of 0.80 when using a two-sided test with size 0.05. Four instances of interim monitoring were planned.

The primary study endpoint was event-free survival (EFS) defined as the time from entry into the study until the occurrence of an event (disease progression, second malignant neoplasm, or death) or until the last contact with the patient, whichever came first. Patients who did not experience an event by the time of last contact were considered censored for EFS-event. The method of Kaplan and Meier was used to estimate the probability of an event as a function of time since enrollment. Equality of risk for EFS-event across the various *NR0B1* GGAA-microsatellite haplotypes was assessed using the log-rank test [22]. All p-values are calculated using the chi-squared approximation and are therefore two-sided. EFS was assessed separately in males and females. Patient sex was not associated with risk for events in AEWS0031 [21].

## Results

### Primary Ewing sarcoma tumor specimens

**Figure 1:**
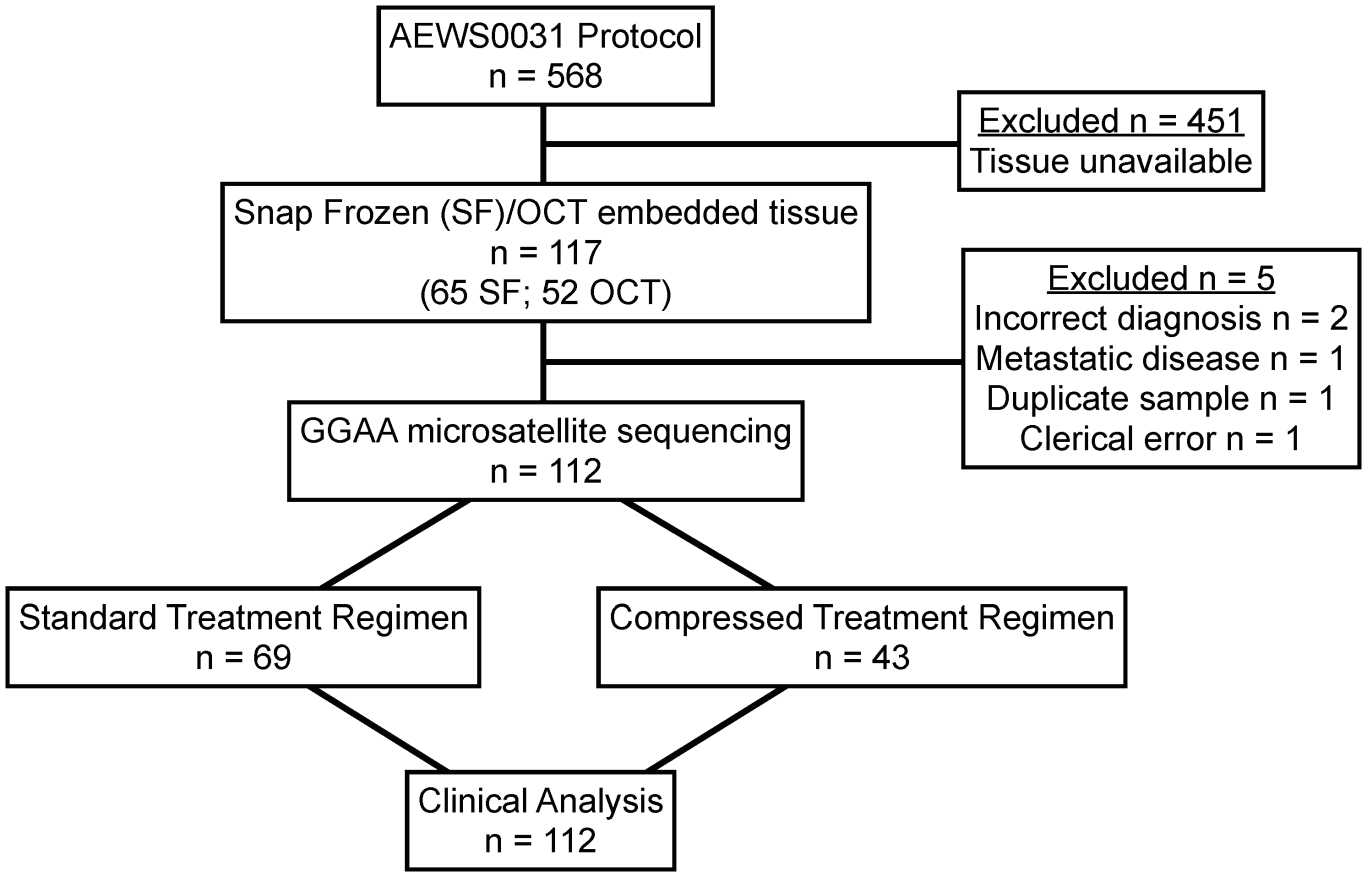
Flow diagram of COG study AEWS0031 patient samples included for GGAA-microsatellite sequencing and clinical analysis.

Ewing sarcoma tissue samples were obtained from the Biopathology Center (Columbus, OH), which serves as the specimen bank for the Children's Oncology Group (COG). All tumor samples were from patients with a pathologically confirmed diagnosis of primary Ewing sarcoma who were enrolled in a large multicenter COG protocol, AEWS0031 [21],[23]. AEWS0031 was opened for enrollment on May 2001 and closed in August 2005. Data current through March 2009 (7.8 years after first enrollment) were used in this analysis. Patients presenting with clinically detectable metastatic disease were excluded. As part of protocol AEWS0031, enrolled patients were prospectively randomized into two different treatment arms: one group received the standard chemotherapy dosing schedule (cycles every 21 days) while the other group received interval compressed dosing (cycles every 14 days) of the same chemotherapeutic regimen, consisting of vincristine (2 mg/m^2^), doxorubicin (75 mg/m^2^), and cyclophosphamide (1.2 g/m^2^) alternating with ifosfamide (9 g/m^2^) and etoposide (500 mg/m^2^). All other study protocols were standardized. Of 568 patients enrolled in AEWS0031, snap frozen (0.004–0.06 gram) or optimal cutting temperature (OCT) compound-embedded tissue (50–60 micron thickness) was available from 117 patients. Of this group, 5 patients were excluded: one patient was represented in duplicate, two patients were determined to have a final tissue diagnosis other than Ewing sarcoma, one patient presented with metastatic disease and one patient could not be properly identified due to a presumed clerical error. The final analytic data set included 112 patients (Figure 1). Ninety-percent (101/112) of patients were identified as Caucasian and only 2% (2/112) were identified as African American. The demographic characteristics of the included and excluded AEWS0031 patients were comparable (Table 1).

**Table 1:** Patient demographics of included and excluded AEWS0031 patients.

	Evaluated Patients (n = 112)		Not Evaluated (n = 456)		p value*
Demographic	n	%	n	%	
**Age**					0.04
<9	38	34%	124	27%	
10–17	68	61%	271	59%	
>17	6	5%	61	14%	
**Sex**					0.8
Male	62	55%	246	56%	
Female	50	45%	210	46%	
**Race**					0.9
White European	101	90%	401	88%	
African American	2	2%	12	3%	
Other	3	3%	19	4%	
Missing	6	5%	24	5%	
**Primary Tumor Site**					0.4
Appendicular	44	39%	151	33%	
Thoracic	16	14%	73	16%	
Pelvic	20	18%	70	15%	
Other Axial	9	8%	66	14%	
Extraosseous	23	21%	96	21%	

*Fisher's exact test.

### The *NR0B1* GGAA-microsatellite is highly polymorphic in Ewing sarcoma tumors and significantly different than white European controls

We have previously evaluated the polymorphic spectrum of three GGAA-microsatellite containing direct EWS/FLI targets: *NR0B1*, *CAV1* and *GSTM4* [12]. The GGAA microsatellites at these loci are polymorphic in human populations, although *NR0B1* was the most polymorphic loci with significant differences observed between African and Caucasian populations. Given the markedly different incidence of Ewing sarcoma in these populations and the role of the NR0B1 protein in sustaining the oncogenic phenotype of Ewing sarcoma, we elected to focus on *NR0B1* for this study. The *NR0B1* GGAA-microsatellite, chrX:30328826 to chrX:30329008 (http://genome.ucsc.edu/cgi-bin/hgTracks; GRCh37/hg19) was amplified, cloned and sequenced in all 112 primary tumor samples. A subcloning strategy was used to sequence all microsatellites, ensuring in heterozygous patients that both alleles were accurately identified. A total of 143 haplotypes were identified, which was expected given 45% of the 112 patients were female. Sequence data were compared to a previously established data set of healthy African and white European controls [12]. It should be noted that in AEWS0031, white, non-Hispanic patients were classified as *Caucasian* and in the aforementioned data-set by Beck et al. [12], white, non-Hispanic subjects of northern European decent are referred to as *European*. For the purpose of clarity, in the present report all white, non-Hispanic patients are reported as *white Europeans*.

**Figure 2:**
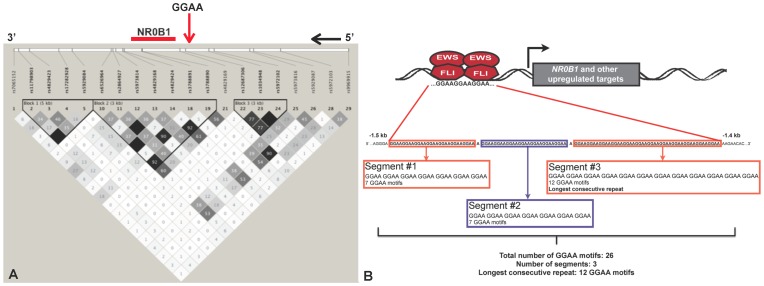
GGAA-microsatellite organization at the *NR0B1* locus. (**A**) Using available single nucleotide polymorphism (SNP) data from the CEU reference population (northern and western European decent) of the International HapMap Project [24], the *NR0B1* GGAA-microsatellite is identified within a defined haplotype block. (**B**) For the *NR0B1* locus, the GGAA-microsatellite is located approximately 1.5 kb upstream of the transcriptional start site (TSS) and is characterized a variable number of contiguous GGAA motifs, partitioned by single adenosine base substitutions. Sequence characteristics of interest include the total number of GGAA motifs, the total number of contiguous segments and longest consecutive GGAA segment. Figure panel adapted from [12].

The *NR0B1* GGAA-microsatellite is located within the promoter region, roughly 1.5 kb upstream of the transcriptional start site. This polymorphic microsatellite ranges in length from 80–240 bp and is located within a defined haplotype block (International HapMap project [24], CEU reference population; Figure 2A). The GGAA-microsatellite is characterized by a series of contiguous GGAA motifs partitioned by a single adenosine base substitution (Figure 2B). Variability exists not only in the total number of GGAA motifs, but also in the number of contiguous segments and the number of GGAA motifs in each contiguous segment. In Ewing sarcoma tumors, *NR0B1* microsatellites ranged in size from small, two-segment repeats containing 16 GGAA motifs to larger multisegment repeats containing up to 61 GGAA motifs. The most frequent haplotype observed in tumor samples was an intermediate sized, 3-segment microsatellite containing 24 GGAA motifs. A comparison of the pertinent microsatellite sequence characteristics in tumor samples and control white European and African populations is presented in Table 2. The descriptive statistical analyses demonstrate that the mean values for total number of GGAA motifs and longest consecutive GGAA segment in the tumor dataset were similar to that of the African data set. The white European dataset had lower mean values for the total number of GGAA motifs compared to both Africans and tumors. Raw sequence data of all included subjects is listed in Table S1.

**Table 2:** NR0B1 GGAA-microsatellite sequence characteristics in Ewing sarcoma tumors and healthy controls.

	Average total number of GGAA motifs*	Most common number of GGAA motifs	Average longest consecutive GGAA segment*	Most common longest consecutive GGAA segment
Ewing sarcoma tumor samples	30±14	24	11±1	10
	Range: 16–61		Range: 8–16	
White European	24±11	24	11±1	11
	Range: 16–60		Range: 8–16	
African	32±15	24	12±1	11
	Range: 14–72		Range: 8–21	

*Mean values ± standard deviation.

**Figure 3:**
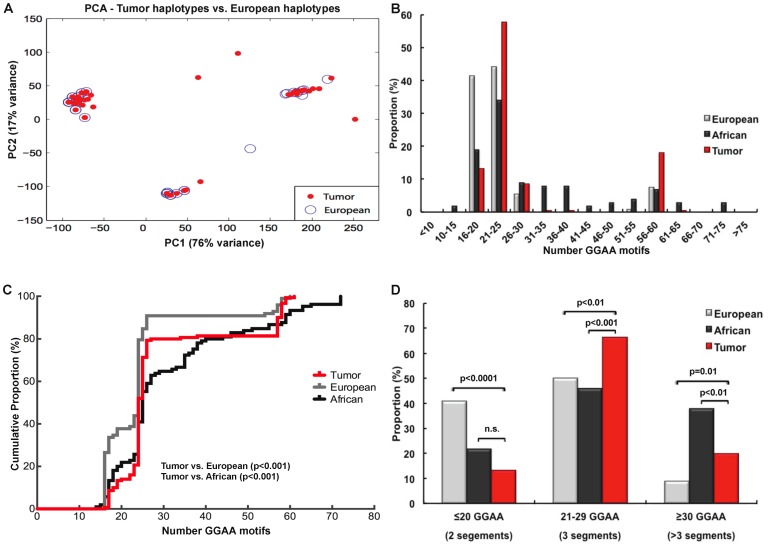
*NR0B1* GGAA-microsatellites are polymorphic in Ewing sarcoma tumors with an allelic distribution different than that of white European and African controls. (**A**) Principal components analysis comparing unique microsatellite haplotypes in tumor samples and white European controls demonstrate three principal sequence clusters, with a high-degree of overlap between the two populations. (**B**) Histogram plots comparing the distribution frequency of *NR0B1* GGAA-microsatellite haplotypes in tumors and white European and African controls. Despite the overlapping PCA analysis, an enrichment of haplotypes containing 21–25 and 56–60 GGAA motifs was observed in tumor samples. Relative to white Europeans, a depletion of haplotypes containing 16–20 GGAA motifs was also noted in tumors. (**C**) Cumulative density plots for each study population similarly demonstrate the enrichment of haplotypes containing 21–25 and 56–60 GGAA motifs in tumors. The distribution of these haplotypes in tumors is significantly different from both white Caucasian and African populations (KS test, p<0.001). (**D**) Stratifying haplotypes according to the major sequence types identified in the PCA demonstrates that intermediate (3 segment) GGAA-microsatellites are more enriched in tumors and larger multisegment haplotypes (>3 segments) were also more enriched compared to white Europeans, although markedly less than Africans. Control white European and African population data from [12].

Given that 90% of the Ewing sarcoma patients analyzed in this study were white Europeans, we sought to determine if the spectrum of *NR0B1* GGAA-microsatellite haplotypes in tumor samples were similar to a previously established control white European data set. To assess this a principal components analysis (PCA) was performed combining the raw *NR0B1* GGAA-microsatellite sequences from both tumor and control white European data sets (Figure 3A). Repetitive regions of each sequence were manually aligned and GGAA repeat motifs were anchored by the single nucleotide adenosine residues that partition the contiguous GGAA repeat units observed in the largest haplotypes. For each GGAA track, the number of GGAA repeats units were counted. The counts of base differences between the flanking non-repetitive regions and the consensus sequence were also determined (gap weight = 0.25). Using this analysis, three distinct haplotype clusters were observed in tumors, which closely overlapped the distribution of haplotypes observed in the white European control data set.

In contrast to the descriptive values reported in Table 2 and the PCA analysis, which examined relationships between unique haplotypes, when the frequency of GGAA-microsatellite haplotypes was plotted as a function of the total number of GGAA motifs, striking differences were observed (Figure 3B). Most notably, a strong enrichment for haplotypes containing 21–25 GGAA motifs was observed in the tumor population: 81/143 (58%) of tumor haplotypes, compared to 46/104 (44%) and 36/106 (34%) white European and African haplotypes, respectively, contained 21–25 GGAA motifs (p = 0.03 and p<0.001, Chi-square), respectively. A second enrichment was also observed for tumor haplotypes containing 56–60 GGAA motifs: 27/143 (19%) of tumor haplotypes, compared to 8/104 (8%) and 7/106 (7%) of white European and African haplotypes, respectively (p = 0.03 and p = 0.01, Chi-square). Additionally, relative to white European controls, a depletion of haplotypes containing 16–20 GGAA motifs was also observed in tumors. The enrichment of tumor haplotypes containing 21–25 GGAA motifs, and the depletion of haplotypes with 16–20 GGAA motifs, contributes to the similar descriptive statistics shown in Table 2, despite the statistically different distribution of these data when more sophisticated techniques are used.

To circumvent some of the inherent bias associated with the arbitrary binning of data, a cumulative density function was performed for each population (Figure 3C). This figure recapitulates the trends observed in Figure 3B, showing a strong enrichment of haplotypes containing 23–26 GGAA motifs in tumor samples, while the European density function is represented by a larger shoulder at smaller GGAA haplotypes (16–20 GGAA motifs) and a similar, although lower amplitude peak in the 23–26 GGAA range. The African density curve is more diffusely populated throughout the spectrum of GGAA motifs. Using a Kolmogorov-Smirnov test [25] to evaluate the haplotype distributions based on the total number of GGAA motifs across all three populations, the tumor data set was statistically dissimilar from both white European (p<0.001) and African (p<0.001) populations (Figure 3C).

Using a slightly different approach, sequence data from all three populations was stratified based on the 3 major haplotype categories identified in the PCA analysis: 2 segment repeats with ≤20 GGAA motifs, 3 segment repeats with 21–29 GGAA motifs and a larger segmental repeats (4–8 segments) with ≥30 GGAA motifs (Figure 3D). Relative to both white European and African control populations, haplotypes containing 21–29 GGAA motifs were statistically over-represented, while haplotypes ≤20 GGAA motifs were under-represented in the tumor population (p = 0.03 and p<0.0001, respectively). These data demonstrate that the distribution of polymorphic *NR0B1* GGAA-microsatellite haplotypes in tumor samples were markedly different than white European populations, which is compelling given the higher incidence of Ewing sarcoma in white non-Hispanic patients of European descent. Such observations may represent a previously unidentified pattern of genetic susceptibility in Ewing sarcoma.

### GGAA-microsatellites are genomically stable throughout oncogenesis and after whole genome amplification

Given the non-overlapping distribution of the *NR0B1* GGAA-microsatellite haplotypes in tumor samples compared to white European controls, we sought to determine if this difference could be attributable to microsatellite instability during the process of oncogenic transformation. Microsatellite instability has been observed in various other cancers, including sarcomas, although most commonly occurring at mono- and dinucleotide microsatellite loci [26]–[28]. To address this question, genomic DNA was extracted from 20 locally-archived primary or metastatic Ewing sarcoma FFPE tissue blocks, and the *NR0B1* GGAA-microsatellite sequence characteristics were compared to matched germ line DNA isolated from bone marrow aspirates. There was no evidence of microsatellite instability in any sample (Table 3). Microsatellite DNA stability is inversely proportional to the length of the microsatellite tract [10] and therefore it was important to assess the stability of the larger *NR0B1* GGAA haplotypes. There was no evidence of microsatellite instability in any of the haplotypes containing 55–60 GGAA motifs (n = 4).

**Table 3:** Comparison of germline and tumor *NR0B1* GGAA-microsatellites.

Patient ID	Tumor	Germline GGAA	Tumor GGAA	Alignment
EWS 17	Metastatic	25/57	25/57	Concordant
EWS 19	Primary	17/25	17/25	Concordant
EWS 22	Primary	25	25	Concordant
EWS 24	Primary	17	17	Concordant
EWS 29	Primary	24	24	Concordant
EWS 36	Primary	25	25	Concordant
EWS 41	Metastatic	25	25	Concordant
EWS 43	Metastatic	24	24	Concordant
EWS 44	Metastatic	25	25	Concordant
EWS 45	Metastatic	24	24	Concordant
EWS 46	Primary	17	17	Concordant
EWS 58	Primary	24	24	Concordant
EWS 59	Primary	24	24	Concordant
EWS 61	Primary	25/57	25/57	Concordant
EWS 62	Primary	24	24	Concordant
EWS 106	Primary	24	24	Concordant
EWS 107	Primary	58	58	Concordant
EWS 115	Primary	23	23	Concordant
EWS 116	Primary	57	57	Concordant
EWS 119	Primary	24	24	Concordant

**Figure 4:**
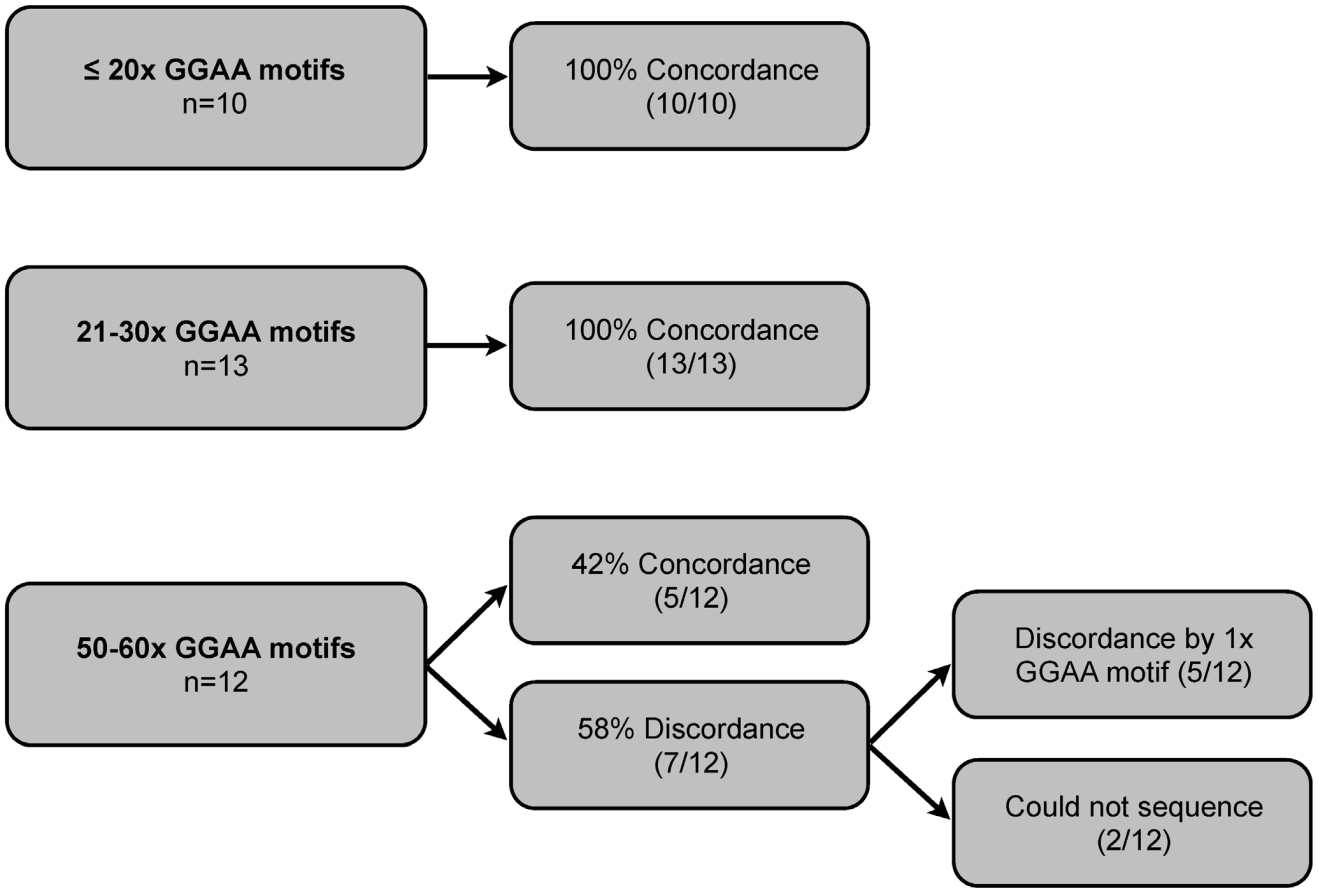
GGAA-microsatellites sequence characteristics after whole genome amplification (WGA). Microsatellites were sequences after WGA and compared to unamplified DNA.

In a complementary series of experiments, we also sought to determine if the process of whole genome amplification (WGA) altered the composition of these GGAA-microsatellites. Given the limited availability of tumor tissue, relatively small reserves of DNA are available for molecular studies in Ewing sarcoma; WGA provides an opportunity to amplify DNA from precious biological samples. Genomic DNA from all 112 Ewing sarcoma samples was commercially amplified using Qiagen's Repli-g WGA service (Qiagen Genomic Services, Hilden, Germany) and GGAA-microsatellite characteristics were compared to unamplified DNA. Repli-g WGA utilizes multiple displacement amplification technology and provides a highly unbiased and complete coverage of the genome [29]. A minimum of 10 WGA amplified tumor samples with an *NR0B1* GGAA-microsatellite sequence for each major sequence category (<20, 20–30, 50–60 GGAA motifs) were sequenced and compared to the unamplified, original DNA source. GGAA-microsatellite sequences were unaltered by the WGA process in 10/10 (100%) and 13/13 (100%) of small (<20 GGAA motifs) and medium (20–30 GGAA motifs) microsatellites, respectively. In the largest microsatellites (50–60 GGAA motifs), sequences were a perfect match in only 4/12 (42%) cases. However, of the 5/7 discordant cases, the WGA sequence was incorrect by only a single GGAA motif. In 2/12 samples, the WGA product did not yield a sequencable amplicon (Figure 4). These data suggest that for small and medium sized GGAA-microsatellites, the WGA process yields highly concordant sequences, although it introduces minor sequence perturbations in larger repeats containing 50–60 GGAA motifs.

### A narrow range of GGAA motifs facilitates maximal EWS/FLI-mediated gene expression

Based on evidence from earlier studies, which initially characterized GGAA-microsatellites as EWS/FLI-response elements, it appeared that after a critical threshold of 4 GGAA motifs, DNA binding and subsequent *NR0B1* gene expression markedly increased with an increasing number of GGAA motifs. However, more recent data has demonstrated the polymorphic spectrum of these GGAA-microsatellites is well beyond the range tested in these earlier biochemical studies. To assess the potential length-dependent relationship between EWS/FLI-mediated gene expression and GGAA-microsatellite polymorphisms, various polymorphic GGAA sequences identified in control populations ranging from 17–72 GGAA motifs were cloned into a luciferase reporter vector directly upstream of the SV40 minimal promoter element. 293 EBNA cells were co-transfected with the various experimental GGAA plasmids and a vector containing EWS/FLI or an empty vector control. All experiments were performed in triplicate and the luciferase data presented is a composite of two independent experiments.

**Figure 5:**
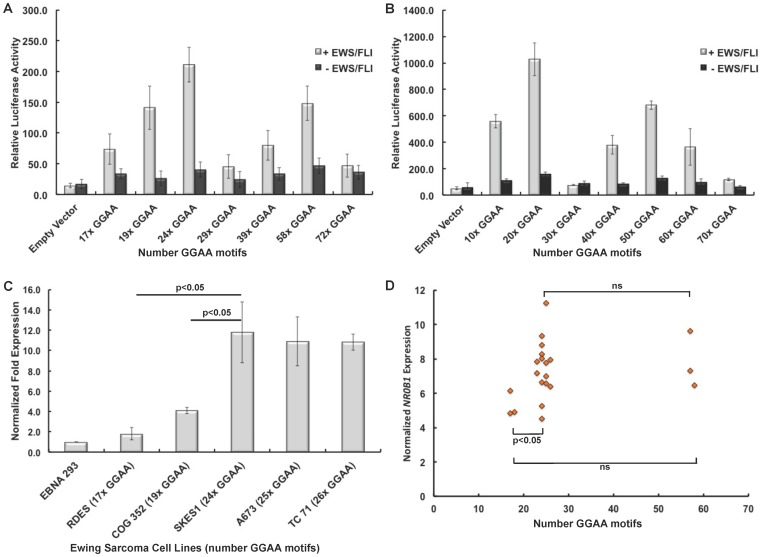
EWS/FLI-mediated gene expression is highly variable across various GGAA-microsatellite length polymorphisms. (**A**) Polymorphic *NR0B1* GGAA-microsatellites from white European and African subjects were cloned into luciferase reporter vectors and co-transfected with EWS/FLI into 293 EBNA cells. A bimodal pattern of gene expression was observed, with greatest expression in constructs with 24 GGAA motifs and a lesser peak in constructs with 58 GGAA motifs. (**B**) A similar bimodal trend was observed using synthetic GGAA constructs identically cloned into the same luciferase reporter construct. (**C**) In patient derived Ewing sarcoma cell lines, RT-PCR quantified *NR0B1* mRNA expression was also maximal in cell lines containing an *NR0B1* microsatellite containing 24–26 GGAA motifs. (**D**) In primary Ewing sarcoma tumors, normalized *NR0B1* transcript levels were lowest in tumors with *NR0B1* GGAA-microsatellites containing 17–18 GGAA motifs, which was significant less than tumors with microsatellites containing 23–26 GGAA motifs (p = 0.04).

In human-derived sequences (Figure 5A), a bimodal relationship of EWS/FLI-mediated gene expression across the spectrum of GGAA constructs investigated was observed. Gene expression was maximal in microsatellites containing 20–25 GGAA motifs, and values precipitously dropped in constructs ranging from 29–40 GGAA motifs followed by a second lesser peak in constructs ranging from 50–60 GGAA motifs. Relative to the 24 GGAA construct, the reduction in expression was maximal in constructs containing 17, 29 and 72 GGAA motifs (3-fold, 4.5-fold and 4.5-fold; p<0.0001), respectively. Expression levels using the 58 GGAA construct were 1.5-fold less than the 24 GGAA construct (p<0.001).

The human-derived sequences contained varying combinations of single-base insertions and contiguous GGAA sequences, thus complicating the interpretation of these data (see Figure 2B). To focus the evaluation on the overall length of the GGAA-microsatellite, synthetic GGAA-microsatellites constructs were synthesized, ranging from 10–70 contiguous GGAA motifs and cloned into the same luciferase vector in an identical fashion. Similar differences were observed in the assays using the synthetic constructs (Figure 5B). Additionally, average gene expression levels in the assays using the contiguous synthetic constructs were markedly elevated compared to the segmental constructs cloned from human DNA (458±330 vs. 107±62, respectively, p = 0.02). These trends suggest contiguous GGAA-microsatellites afford more optimal gene expression than partitioned repeats. Exemplifying this, gene expression in the smallest synthetic construct (10 GGAA) was 2.5-fold greater than the maximal expression observed from the partitioned 24 GGAA construct. Interestingly, the third segment of the 24 GGAA construct contains 10 contiguous GGAA motifs.

To assess the influence of these polymorphic GGAA-microsatellite response elements in a more native cellular context, *NR0B1* mRNA levels were quantified from various patient-derived Ewing sarcoma cell lines confirmed to be polymorphic at the *NR0B1* GGAA locus. Given the position of *NR0B1* on the X chromosome, to circumvent any issues associated with heterozygosity and potential X-linked inactivation, only cell lines either homozygous or hemizygous for a polymorphic *NR0B1* GGAA-microsatellite locus were included. Unfortunately none of the investigated cell lines were hemizygous or homozygous for a larger 50–60 GGAA motif allele; two cell lines (EWS502 and TC32) were heterozygous (20/58 GGAA and 24/58 GGAA, respectively), but a clear pattern of allelic activation (or inactivation) could not be established for these cell lines. Figure 5C illustrates quantitative RT-PCR normalized expression levels of *NR0B1* mRNA transcripts relative to the number of GGAA motifs measured. Similar to the luciferase experiments, maximal gene expression was observed in cell lines ranging from 24–26 GGAA motifs. Negligible *NR0B1* levels were observed in the RDES cell line, which is hemizygous for a 17 GGAA-microsatellite. These results using a native cellular context strongly support the trends observed in both patient-derived and synthetic luciferase experiments of maximal gene expression in constructs containing 20–25x GGAA motifs.

When comparing the gene expression profiles (Figure 5) to the allelic distributions of the *NR0B1* GGAA-microsatellite sequenced from tumor samples (Figure 3), striking similarities are observed. Notably, the bimodal pattern of maximal gene expression and the amplitude of these peaks in constructs ranging from 20–25x and 56–60x GGAA motifs parallels the frequency and distribution of *NR0B1* GGAA-microsatellite in tumors.

To investigate if GGAA-microsatellite polymorphisms influenced *NR0B1* gene expression in Ewing sarcoma tumors, we quantified normalized *NR0B1* expression using microarray data from 31 Ewing sarcoma samples from which both PCR sequencing data and RNA were available. Ten of the 31 samples were heterozygous at the *NR0B1* GGAA-microsatellite locus, leaving 21 hemi- or homozygous tumors for analysis (Figure 5D). Consistent with the cell line data in Figure 5C, we observed lower normalized *NR0B1* expression levels in tumors containing only 17–18 GGAA motifs in their *NR0B1* microsatellite. This was a statistically-significant diminished level as compared to tumors containing 23–26 GGAA motifs (p = 0.04). Thus, the human tumor data is consistent with the cell line and *in vitro* luciferase studies.

### Polymorphisms of the *NR0B1* GGAA-microsatellite are not predictive of event free survival

Given the documented influence of GGAA length polymorphism on gene expression and mRNA levels, we next sought to determine if these polymorphisms influence tumor biology and clinical outcomes in patients with Ewing sarcoma. *NR0B1* is one the most highly upregulated, direct EWS/FLI targets, and expression of this gene is essential for transformation in Ewing sarcoma cell lines [6], [30]. Clinical outcome data for at least 5 years (in surviving patients) [21] was available in all 112 samples used in the sequencing analysis. Of the 112 patients included in the analysis from AEWS0031, 69/112 were treated with the standard chemotherapy regimen as compared to 43/112 treated with compressed chemotherapy (Figure 1). It should be noted that the 5-year EFS was slightly improved in patients receiving compressed therapy (73% vs. 65%, p = 0.048 [21]). The aggregate outcome of patients who were considered in this analysis was similar to patients who were eligible for AEWS0031 but who were not included in the analysis (p = 0.21).

**Figure 6:**
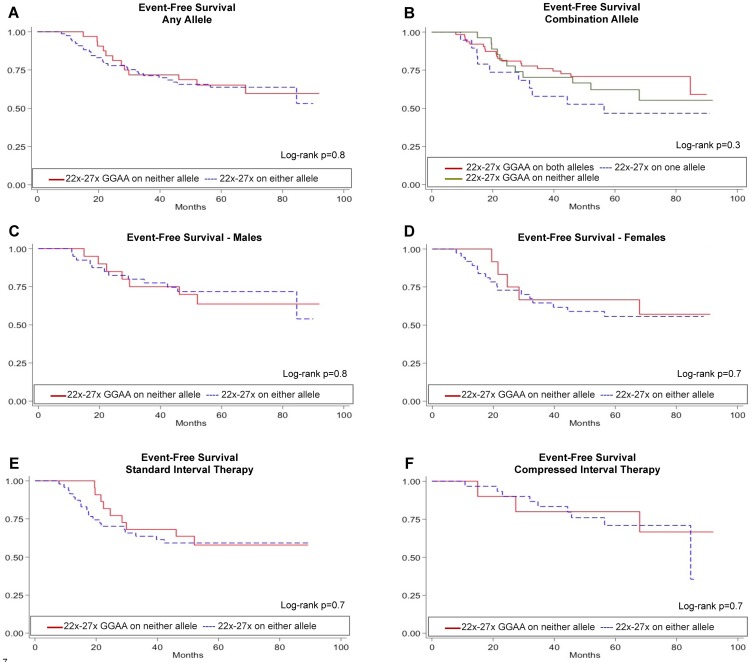
*NR0B1* GGAA-microsatellite polymorphisms do not influence event free survival (EFS) in Ewing sarcoma patients. (**A**) EFS was compared in 112 patients from AEWS0031 based on the presence of absence of at least one *NR0B1* GGAA-microsatellite allele containing 22–27 GGAA motifs. This allele type was chosen based on the pattern of alleles present in tumor samples and the maximal EWS/FLI-mediated gene expression supported by alleles of this length category. (**B**) EFS was similarly assessed based on the presence of one or both alleles containing 22–27 GGAA motifs. Additional subgroup analyses were also performed in males (**C**) and females (**D**) and in patients receiving standard (**E**) or compressed (**F**) therapy.

Given the biochemical data favoring optimal gene expression over a narrow range of GGAA motifs and the distribution of these haplotypes in Ewing sarcoma tumors, Kaplan-Meier survival analyses were performed stratifying patients based on the presence or absence of *NR0B1* alleles containing 22–27 GGAA motifs (Figure 6). The presence of one or more *NR0B1* alleles containing 22–27 GGAA motifs did not influence EFS compared to patients without these alleles (Figure 6A and 6B). Given that females represented 45% of our cohort, numerous patients heterozygous for different length alleles were identified. Whether one or both of these alleles is active in tumor cells remains unclear; therefore, EFS was assessed separately in males and females (Figure 6C and 6D). In male and female patients, EFS was also not influenced by allele type. Furthermore, stratifying the patients based on assignment to standard vs. compressed chemotherapy arms also did not influence EFS survival based on allele type (Figure 6E and 6F). These results clearly demonstrate that despite biochemical data showing a strong relationship between GGAA-microsatellite length and gene expression levels, polymorphisms of the *NR0B1* GGAA-microsatellite do not influence clinically relevant outcomes.

## Discussion

Transcriptional dysregulation via microsatellite DNA in Ewing sarcoma represents a fascinating and novel property of the EWS/FLI chimera. Microsatellite DNA is not subject to the same evolutionary pressures as coding DNA, rendering these sequences highly polymorphic across individuals and populations [11], [12], [31]. Furthermore, given that 40–50% of genomic EWS/FLI occupancy occurs at GGAA-microsatellites [7], these EWS/FLI-responsive elements provide a unique opportunity to examine Ewing sarcoma susceptibility and pathogenesis from an alternative genetic basis. In particular, our research group is interested in whether polymorphisms at transcriptionally important GGAA-microsatellites are biologically relevant in this context. In the present study, we have demonstrated that the *NR0B1* GGAA-microsatellite in primary Ewing sarcoma tumors is highly polymorphic, with an allelic distribution dissimilar from white European controls. Here we report the first series of biochemical experiments detailing the effect of GGAA-microsatellite polymorphisms on EWS/FLI-mediated transcriptional regulation demonstrating that the distribution of these *NR0B1* haplotypes in tumors is strongly biased towards a narrow range of microsatellite alleles that facilitate maximal EWS/FLI-mediated gene expression.

Traditionally viewed as “junk” DNA, microsatellite DNA is becoming increasingly recognized as an important cis-regulating genetic element [32], [33]. The discovery of GGAA-microsatellites as a direct EWS/FLI-mediated transcriptional response element in Ewing sarcoma identified a novel function of microsatellite DNA in human cancer development and a previously unrecognized ETS factor binding site [34]. We have demonstrated that across a large numeric range of GGAA motifs, EWS/FLI-mediated gene expression is highly variable. However, contrary to our preliminary understanding of these EWS/FLI-responsive elements, we did not observe a simple linear relationship of increasing gene expression as a function of an increasing number of GGAA motifs [5]. Instead, a bimodal relationship was observed. A mechanistic explanation for this bimodal relationship was not assessed in the present study, although similar findings have been observed in other model systems. For instance, in *Neisseria meningitides*, expression of a virulence factor, *NadA* is regulated by a polymorphic, promoter-based tetranucleotide microsatellite element with a similar pattern of transcript periodicity to that observed in our study [35], [36]. The variations in *NadA* transcript levels were attributed to altered binding abilities of transcriptional cofactors across the various microsatellite polymorphisms [36].

The EWS/FLI chimera requires a minimum of 4 contiguous GGAA motifs (16 bp) to effectively bind microsatellite DNA. Furthermore, EWS/FLI occupies these microsatellites in a ratio of 2 protein molecules for every DNA molecule in synthetic microsatellite constructs comprised of 4, 5, 6 and 7 contiguous GGAA motifs [9]. A potential explanation for the bimodal biochemical expression patterns observed in this study is that the stoichiometric occupancy of EWS/FLI and associated co-factors is most optimal across microsatellites containing 21–25 or 55–60 GGAA motifs. Another possibility is that certain GGAA-microsatellite polymorphisms are more (or less) likely to form inhibitory secondary DNA structures. Guanine-rich DNA sequences can predispose to the formation of non-B-form DNA structures and G-quadruplexes [32], [37], which may influence EWS/FLI and associated co-factor occupancy. Certainly, the results of the luciferase, cell line, and primary human tumor data detailed in the present study are compelling and warrant further investigations into the biochemical effects of GGAA content on EWS/FLI-mediated DNA binding in a native cellular and chromatin context.

The incidence of Ewing sarcoma in African populations is 10-fold less than that of white Europeans [13], but as of yet there is no concrete explanation for this difference [38]–[40]. The GGAA-microsatellite of two critical upregulated EWS/FLI-targets in Ewing sarcoma (*NR0B1* and *CAV1*) have been shown to be highly polymorphic in African and white European populations, with a predisposition for significantly larger GGAA-microsatellites in Africans, especially at the *NR0B1* locus [12]. This finding prompted further inquiry into the makeup of these elements in Ewing sarcoma tumor samples. Indeed, our results demonstrate that these GGAA elements are highly polymorphic in tumors, although the distribution of these haplotypes within primary tumors demonstrated compelling differences compared to both African and white European controls. The dissimilar distribution of tumor and white European control haplotypes is an important observation, given that 90% of the AWES0031 cohort was identified as white European. Our preliminary hypothesis was that the *NR0B1* GGAA-microsatellite sequence data set from white European controls would be very similar to the patient-derived tumor samples.

Compared to white European controls, a strong enrichment for *NR0B1* microsatellite haplotypes containing either 21–25 or 56–50 GGAA motifs and a bias against smaller alleles containing 17–20 GGAA motifs was observed in patient samples. Given the stability of these GGAA sequences as determined by the comparison of tumor and germline DNA sequences, the predilection for those two allele ranges does not appear to be a product of sequence evolution within tumor cells during the process of oncogenesis. Given that *NR0B1* is among the most upregulated direct EWS/FLI targets, and is essential for maintenance of oncogenic transformation [5], [6], [14], two alternative hypotheses were proposed: the predilection for the selection bias of specific *NR0B1* GGAA-microsatellite haplotypes in tumors is a consequence of either superior oncogenic potential in tumors harboring 21–25 or 56–50 GGAA motifs at the *NR0B1* locus or conversely, these principal GGAA-microsatellite haplotypes observed in tumors are important for Ewing sarcoma susceptibility and transformation in progenitor cells harboring the EWS/FLI translocation. The luciferase assays and qRT-PCR data from human-derived cell lines clearly show that the most common GGAA-microsatellite allele observed in tumors also facilitates maximal EWS/FLI-mediated gene expression. The results from these experiments are further supported by patterns of *NR0B1* gene expression observed in tumor microarray data, wherein tumors harboring small GGAA microsatellites (<20 GGAA repeats, Figure 5D) are those that have the lowest levels of *NR0B1* gene expression. Although interpretation of tumor microarray experiments is limited by the small number of samples included, the limited number of samples available with smaller numbers of GGAA repeats further supports the hypothesis that Ewing sarcoma tumor development is restricted by lower levels of *NR0B1* expression in the setting of these small GGAA-microsatellites. Thus, these results provide additional supportive data in a more biologically relevant context. Likewise, the clinical analysis of AEWS0031 patients demonstrates that EFS is not influenced by these *NR0B1* GGAA polymorphisms, which we believe also supports the latter hypothesis.

Assessing the clinical impact of these *NR0B1* GGAA polymorphisms was an important outcome measure in this study and consequently various statistical approaches were employed to sufficiently address this association. However, when subgroup analyses were performed to address potential confounding influences such as patient sex, zygosity, and chemotherapy our results clearly demonstrate that disease behavior in Ewing sarcoma is not influenced by GGAA-microsatellite polymorphisms at the *NR0B1* locus.

**Figure 7:**
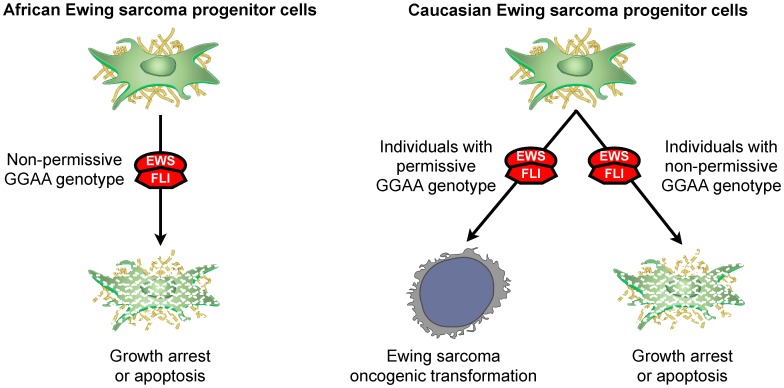
Model of GGAA-microsatellite polymorphism contributions to Ewing sarcoma susceptibility in African and white European populations.

Integrating the results of this study we propose that in Ewing sarcoma, GGAA-microsatellite polymorphisms play an important role in disease susceptibility. It is generally accepted that the EWS/FLI translocation event is the driver oncogenic mutation in Ewing sarcoma. We suggest that in precursor cells exposed to the EWS/FLI chimera, cells with a more ‘permissive’ genetic constitution of GGAA-microsatellite polymorphisms are more likely to transform when exposed to the EWS/FLI chimera than cells with a non-permissive GGAA genotype (Figure 7). Further supporting this model is that Ewing sarcoma is believed by many experts to be exclusively a human condition; spontaneous cases of Ewing sarcoma have not been observed in any other animal species (except for a single case report in a camel [41]), and inducible Ewing sarcoma models in murine progenitor cell and transgenic mice do not recapitulate the molecular hallmarks of disease [42]–[45]. Interestingly, the mouse orthologs of *NR0B1, CAV1, GSTM4, and FCGRT* (4 microsatellite-containing upregulated EWS/FLI targets in humans) do not possess a GGAA-microsatellite in their respective promoter/enhancer regions. Additionally, ectopic EWS/FLI expression in murine-derived NIH3T3 cells does not upregulate *Nr0b1*, further supporting observation that GGAA-microsatellites are necessary for regulation of *Nr0b1* in Ewing sarcoma [46]. Additional sequencing efforts are underway to better characterize a more comprehensive cohort of EWS/FLI-enriched GGAA-microsatellites in African, white Europeans and Ewing sarcoma patients.

In a recent genome-wide association study three candidate Ewing sarcoma susceptibility loci were identified using a comprehensive single nucleotide polymorphism (SNP) analysis [39]. The authors demonstrated a greater frequency of these susceptibility loci in white Europeans as compared to Africans. However, the oncogenic contribution of these identified susceptibility loci in the pathogenesis of Ewing sarcoma has yet to be clarified. Furthermore, it does not appear that the observed differences in the frequency of these susceptibility loci will fully account for the 10-fold increase in Ewing sarcoma in white Europeans compared to Africans. EWS/FLI-responsive GGAA-microsatellites provide a complementary genetic approach to understand these discrepant patterns of disease incidence. These GGAA-microsatellites are highly polymorphic and are also direct genetic targets of the EWS/FLI chimera. Additionally, compared with white European and Asian populations, African populations have are known to have increased genetic diversity for many microsatellite loci [47]. Based on our biochemical data, a greater diversity of GGAA motifs at important microsatellite loci may actually negatively impact EWS/FLI-mediated gene expression, which appears optimal over a narrow range of GGAA motifs. Additional work will be needed to discern the relative contributions of microsatellite polymorphisms and SNPs in the susceptibility to Ewing sarcoma development.

An additional important finding gleaned from this study is that GGAA-microsatellites are genetically stable during the process of oncogenic transformation. Consequently, tumor tissues are not required to obtain DNA for GGAA-microsatellite genotyping in individuals with Ewing sarcoma. Given the current practice of CT-guided core biopsies and neoadjuvant chemotherapy, Ewing sarcoma tissue is infrequently available for genetic studies. Germline sources of DNA such as blood, bone marrow aspirates, saliva and buccal swabs are more readily available and based on the results presented here, can be used in future GGAA-microsatellite genotyping experiments. Additionally, our data also demonstrates that commercial WGA of tumor DNA does not erroneously expand or contract small or medium sized GGAA-microsatellites. Even in extremely large microsatellites (50–60 GGAA motifs) discordance was minimal (1 GGAA motif). Importantly, together these findings provide valuable insight into the stability of GGAA-microsatellites in Ewing sarcoma, providing an opportunity for prospective genotyping studies to progress beyond the barriers of limited tissue supplies.

In conclusion, this report is the first detailed examination of EWS/FLI-responsive GGAA-microsatellite polymorphisms in Ewing sarcoma. At the *NR0B1* locus, we have demonstrated that in primary Ewing sarcoma tumor samples, there is strong overrepresentation of a narrow range of GGAA haplotypes, which was discordant from healthy white European controls. We further demonstrated that maximal EWS/FLI-mediated gene expression is also highly dependent on a comparably narrow range of GGAA motifs. At the *NR0B1* locus, these polymorphisms do not influence clinical outcomes, favoring a model in which these GGAA polymorphisms may contribute to the elusive permissive cellular and genetic environment necessary for EWS/FLI-mediated transformation.

## Supporting Information

Table S1 Complete sequence data of *NR0B1* GGAA-microsatellites form all samples. In the “COG Data” tab all data from Children's Oncology Group primary tumor samples are included. The data fields indicate the zygosity, the number of GGAA repeats for each allele, the segment characteristics of each allele, and the raw sequence of each allele. In the “African Data” and “European Data” tabs, all data from the African and European subjects, respectively, are included. The data from these latter tabs were previously described in reference [12].
(XLSX)
